# Digital Care Program for Urinary Incontinence in Females: A Large-Scale, Prospective, Cohort Study

**DOI:** 10.3390/healthcare12020141

**Published:** 2024-01-08

**Authors:** Dora Janela, Anabela C. Areias, Maria Molinos, Robert G. Moulder, Ivo Magalhães, Virgílio Bento, Marta Cardeano, Vijay Yanamadala, Fernando Dias Correia, Jennesa Atherton, Fabíola Costa

**Affiliations:** 1 Clinical Research, Sword Health Inc., Draper, UT 84020, USA; d.janela@swordhealth.com (D.J.); a.areias@swordhealth.com (A.C.A.); mmolinos@swordhealth.com (M.M.); robertgm111@gmail.com (R.G.M.); vbento@swordhealth.com (V.B.); v.yanamadala@swordhealth.com (V.Y.); fcorreia@swordhealth.com (F.D.C.); 2 Institute for Cognitive Science, University of Colorado, Boulder, CO 80309, USA; 3 Bloom, Sword Health Inc., Draper, UT 84020, USA; ivomagalhaes@swordhealth.com (I.M.); marta@swordhealth.com (M.C.); j.atherton@swordcare.com (J.A.); 4 Department of Surgery, Frank H. Netter School of Medicine, Quinnipiac University, Hamden, CT 06473, USA; 5 Department of Neurosurgery, Hartford Healthcare Medical Group, Westport, CT 06103, USA; 6 Neurology Department, Centro Hospitalar e Universitário do Porto, 4099-001 Porto, Portugal

**Keywords:** women’s health, pelvic floor dysfunction, pelvic floor muscle training, telerehabilitation, digital therapeutics, biofeedback

## Abstract

Female urinary incontinence (UI) is highly prevalent in the US (>60%). Pelvic floor muscle training (PFMT) represents first-line care for UI; however, access and adherence challenges urge new care delivery models. This prospective cohort study investigates the feasibility and safety of a remote digital care program (DCP) combining education and PFMT with real-time biofeedback with an average duration of 10 weeks. The primary outcome was the change in the Urinary Impact Questionnaire—short form (UIQ-7) from baseline to program-end, calculated through latent growth curve analysis (LGCA). Secondary outcomes included the impact of pelvic conditions (PFIQ-7), depression (PHQ-9), anxiety (GAD-7), productivity impairment (WPAI), intention to seek additional healthcare, engagement, and satisfaction. Of the 326 participants who started the program, 264 (81.0%) completed the intervention. Significant improvement on UIQ-7 (8.8, 95%CI 4.7; 12.9, *p* < 0.001) was observed, corresponding to a response rate of 57.3%, together with improvements in all other outcomes and high satisfaction (8.9/10, SD 1.8). This study shows the feasibility and safety of a completely remote DCP with biofeedback managed asynchronously by a physical therapist to reduce UI-related symptoms in a real-world setting. Together, these findings may advocate for the exploration of this care delivery option to escalate access to proper and timely UI care.

## 1. Introduction

Urinary incontinence (UI) is experienced by more than 60% of women in the United States (US), with increasing prevalence and symptom severity per each decade of life [1]. UI impacts all aspects of patients’ daily lives—from mental health to sexuality to social and work life [2]. Productivity is extensively affected, frequently demanding job changes or leading to job resignations [3]. The socioeconomic impact of UI is undeniable, with annual overall costs in 2007 reaching USD 65.9 billion for overactive bladder with UI alone [4].

Pelvic floor muscle training (PFMT) represents first-line care for UI [5,6], being widely established as a treatment for stress UI and mixed UI and less studied for urgency UI [7]. Clinical guidelines recommend PFMT combined with education and lifestyle modification advice [5,6]. Despite these recommendations, access to PFMT is blocked by multiple healthcare and individual barriers [8,9,10,11]. Challenges in surpassing geographical and time constraints to access healthcare facilities are aggravated by the scarcity of trained healthcare providers to adequately supervise and guide PFMT [11]. Moreover, unsupervised home therapy has been frequently reported as an ineffective solution due to low adherence levels and poor knowledge about proper technique, frequency, and duration of training [12,13]. Adding to the aforementioned barriers, the growing prevalence of UI and the taboo, embarrassment, and stigma surrounding these conditions have increased the number of patients without proper and timely treatment [8].

Different digital care delivery strategies have been explored to improve access to and adherence to treatment [14]. Findings from several systematic reviews suggest the benefit of digital interventions in improving UI symptoms [14,15,16,17].

Initial mobile apps for UI were designed to provide instructions to patients through digital, dynamic representations. However, they lacked biofeedback features or healthcare professional involvement [18,19]. The introduction of urotherapists for remote support was attempted by Sjöström et al. [20]. More recently, the incorporation of biofeedback has been reported to promote greater PFMT self-efficacy [21], as well as better adherence and PFMT outcomes [22,23,24,25]. The increasing prevalence of patients reporting UI warrants further exploration of new delivery solutions.

Herein, we report about a completely remote pelvic digital care program (DCP)—the Bloom program—combining education with PFMT using real-time biofeedback—asynchronously managed by a physical therapist. This study aims to explore the feasibility and safety of such a program while comprehensively evaluating the potential of this pelvic DCP in reducing the impact of urinary symptoms on daily living. As a secondary aim, we will evaluate the impact in terms of mental health, work productivity, and the intent to seek additional healthcare. We hypothesized that participants would report improvement in all outcomes after the program.

## 2. Materials and Methods

### 2.1. Study Design

This is a decentralized, prospective single-arm observational cohort study, which was approved by the Advarra Institutional Review Board (Pro00064510) and registered on ClinicalTrials.gov (NCT05513417) on 24 August 2022. The recruitment period was from 9 August 2022 until 18 January 2023, and the home-based DCP was conducted between 27 August 2022 and 22 May 2023.

### 2.2. Participants

Females (>18 years old) who are beneficiaries of employers or health plans covered by the Bloom program from 45 states in the US and the District of Columbia were invited to apply to the study through a dedicated website. Those with self-reporting UI, defined as the complaint of any involuntary loss of urine according to the International Consultation on Incontinence (ICI) [5], were considered. Patients that did not have a previous diagnosis were characterized on the specific UI (i.e., stress, urgency, and mixed) according to the trigger of the leakage: stress when on effort or exertion, on sneezing, or coughing; urgency when accompanied by or immediately preceded by urgency; mixed for the coexistence of both types [5].

Exclusion criteria comprised: (1) inability to perform 20 min of light–moderate exercise; (2) active cancer or under treatment for cancer; (3) surgery, significant trauma, or other conditions where mobilization is contra-indicated; (4) uncleared clinical red flags suggestive of serious underlying conditions; (5) signs of acute, serious neurologic compromise; (6) pelvic infection or suspicion of inflammatory bowel disease; (7) complicated or high-risk pregnancy; (8) contra-indication for vaginal penetration; (9) clinical conditions (e.g., dementia) precluding compliance with autonomous home-based exercise; (10) silicone allergy. When considering an exclusion, the individual presentation and medical clearance (if applicable) were critical to the physical therapist’s eligibility decision.

Informed consent was obtained from all participants. To be considered in the study, the participant had to complete at least three sessions. Participants were considered dropouts in the absence of exercise sessions for 30 consecutive days. Those who did not complete reassessment surveys but were compliant with the intervention were included.

### 2.3. Intervention

The intervention consisted of biofeedback-mediated pelvic floor muscle training (PFMT), functional exercises, and education with an average duration of 10 weeks (Supplementary Table S1).

**Figure 1:**
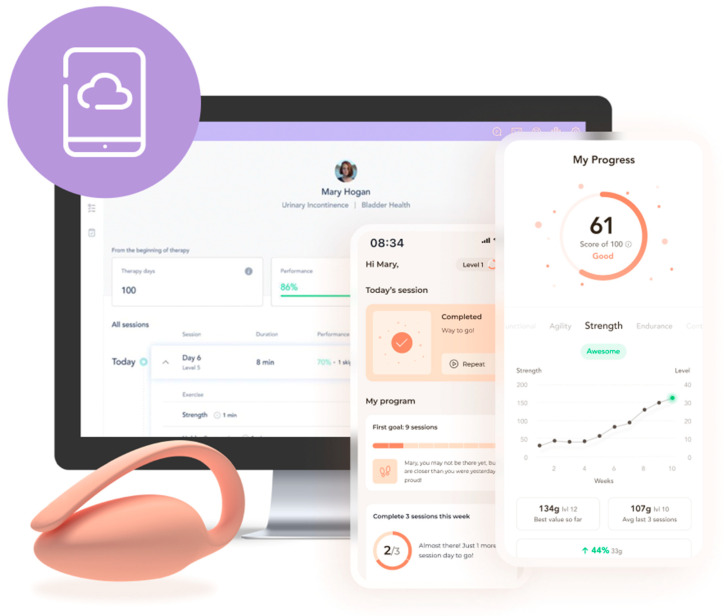
Components of the patient’s medical device: wireless intravaginal sensor, mobile app, and cloud platform.

After enrollment, each participant filled out an onboarding form with demographic and clinical questions (see outcomes below) and chose a physical therapist specializing in pelvic health (PT) responsible for managing and continuously tailoring the intervention. The initial clinical assessment was performed by the PT during the onboarding video call, leveraging the information provided by the patient upon enrollment and the clinical presentation (e.g., the presence of comorbid pelvic floor conditions). Intervention goals were established collaboratively between the PT and the patient. An FDA-listed medical device composed of an intravaginal sensor, a dedicated mobile app (to be downloaded to the patient’s smartphone), and a cloud-based platform was used for remote care (Figure 1). A kit containing the sensor and water-based lubricant was sent to each patient. The intravaginal sensor includes a force transducer to measure PFM activity (both contraction and relaxation) and an accelerometer to measure PFM motion, where a cranial lift was identified during contraction and a caudal descent during relaxation, providing real-time biofeedback to participants. These sensors, located in the pebble-shaped section of the device, connect with the mobile app wirelessly using Bluetooth, located in the tail of the device, which remains outside the patient’s body.

Patients were first educated on the UI condition by the assigned PT during an onboarding video call about PFMT appropriateness, treatment goals, diaphragmatic breathing training (to be performed during exercise sessions), behavioral modifications, how to insert, use, and sanitize the intravaginal sensor, and how to perform the contraction and relaxation of the PFM.

Gamified exercise sessions, along with written instructions, were displayed in the app. The intravaginal sensor provided real-time biofeedback according to the specific contraction and relaxation target defined on the prescription (as a default, three sessions per week were recommended; further details on exercise parameters are in Supplementary Table S1).

Education was delivered through the mobile app throughout the program in the form of written articles following clinical guidelines and research [6] and accompanied by the dedicated PT.

The cloud-based portal stored data on each education content interaction and sensor-related exercise session performance, enabling continuous asynchronous monitoring and tailoring protocol progress by the PT.

### 2.4. Outcomes

Participants completed assessment surveys at baseline, session 9, and session 15. The primary outcome was the change in the Urinary Impact Questionnaire—short form (UIQ-7) from baseline to treatment end. UIQ-7 assesses the impact of urinary-related symptoms in activities of daily living (scored 0–100%, with higher scores indicating worse symptoms) [26], and its use is recommended by ICI [27]. UIQ-7 responsiveness was previously validated [28]. Secondary outcomes included engagement, satisfaction, and the validated questionnaires: Pelvic Floor Impact Questionnaire—short form 7 (PFIQ-7) [26] to assess the impact of bladder, bowel, or vaginal symptoms in daily activities; Work Productivity and Activity Impairment (WPAI) for general health [29]; Generalized Anxiety Disorder 7-item scale (GAD-7) [30]; Patient Health 9-item Questionnaire (PHQ-9) [31] and the question, “How likely are you to seek additional healthcare interventions for this condition?” (0—not at all likely to 10—extremely likely), as depicted in Table 1.

### 2.5. Safety and Adverse Events

Regular and on-demand communication between participants and PT was established through video calls and text messages (built-in secure chat in the mobile app) to provide support and feedback, enhance motivation, and ensure safety. Participants were instructed to report any adverse event by any channel to the dedicated PT. Self-reported symptom and fatigue levels (scores 0–10) were registered following each exercise session and continuously monitored by the PT.

### 2.6. Data Availability

All relevant data are included in the article or available as Supplementary Material Data. De-identified data and analysis codes may be provided upon reasonable request to the corresponding author.

### 2.7. Sample Size

A sample size calculation was performed to detect a small effect size of 0.2 [32] of the mean change in UIQ-7 from baseline to treatment end, considering a mean difference between two dependent means. Assuming an 80% power to detect this change and a two-sided 0.05 significance level, a total of 199 participants were estimated. Allowing for an attrition rate of 20% owing to the uncertainty introduced by the real-world context, a number of participants above 250 were targeted.

### 2.8. Statistical Analysis

Demographic and clinical data and engagement metrics were reported through descriptive statistics, with continuous variables described as mean (standard deviation) and categorical variables as frequencies (percentage). Baseline differences between completers and non-completers (i.e., those who dropped out or were excluded after the program started) were assessed through chi-squared tests for categorical variables and independent sample *t*-tests or one-way ANOVA for continuous variables. Baseline correlations were evaluated using the Pearson correlation.

A response bias analysis was conducted, confirming that the missing data were at random. This was performed by assessing whether no response to reassessments was related to baseline characteristics (including demographics and baseline clinical scores). Considering time as an important factor in the analysis, reassessment data were binned according to the respective time frame in which the reassessment was completed (i.e., 5 and 10 weeks).

Latent growth curve analysis (LGCA), based on a structural equation model, was applied to assess the longitudinal trajectory of clinical outcomes, considering time as a continuous variable. LGCA provides an estimate of the average trajectory and individual variation based on each individual data. This methodology has several benefits, including providing model fit measures and handling missing data through full information maximum likelihood (FIML). FIML uses all available data from all participants independently of missing a specific time point, outperforming other missing data handling methods [33]. A conditional analysis was conducted to assess the influence of age, body mass index (BMI) (continuous variables), and parity (discrete variable) as covariates, fitted as random effects to allow each to vary between individuals. All models were estimated with a robust sandwich estimator for standard errors.

To estimate the response rate to UIQ, the minimal clinically important change (MCIC) of 17 points reported for the UIQ long-form [34] was applied to overcome the lack of UIQ-7 MCIC for non-post-surgical conditions. To that end, UIQ-7 scores were converted to UIQ long-form scores as previously [24,35], followed by a binary logistic regression. Binary logistic regression was also used to estimate the response rate for PHQ-9, GAD-7, and WPAI overall, considering an MCIC of 3.8 [36], 5 [37], and 7 [38], respectively.

All analyses followed an intention-to-treat approach (considering all participants at the program start), and significance levels were set at *p* < 0.05. LGCA was coded through R (version 1.4.1717), and all other analyses were conducted through SPSS (version 17.0, SPSS Inc., Chicago, IL, USA).

## 3. Results

### 3.1. Participants

**Figure 2:**
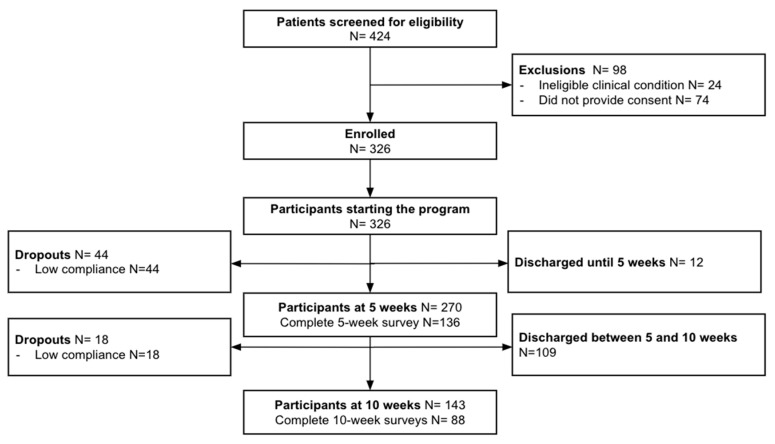
Study flow chart.

From the 424 participants screened for eligibility, 98 declined participation (Figure 2). The program started with 326 participants, of whom 264 completed it, for a completion rate of 81.0%.

### 3.2. Baseline Characteristics

Participant demographics are described in Table 2. The cohort had an average age of 44.8 years (SD 9.1), an average BMI of 29.7 (SD 7.0), a high proportion of participants with obesity (39.3%), and of parous participants (92%), and was diverse, with 21.7% of members from minority races/ethnicities. Most participants had higher education (65.9%) and were full-time employees (84.4%). Additionally, a majority of participants reported urinary symptoms persisting for >6 months (85.6%) and stress UI specifically (73.3%, 239/326). Comparing completers and non-completers (Supplementary Table S2), no significant differences were observed except for average age and BMI, where completers were older (45.3, SD 9.1 vs. 42.7, SD 9.1, *p* = 0.041), had lower BMI levels (29.2, SD 6.7 vs. 31.7, SD 7.9, *p* = 0.009), and had lower PHQ-9 scores among those with at least mild depression at baseline (10.0, SD 4.4 vs. 12.8, SD 5.7, *p* = 0.027).

### 3.3. Clinical Outcomes

The intention-to-treat approach model estimates and respective model fit are provided in Supplementary Table S3, while the effect of covariates on outcome estimates is presented in Supplementary Table S4.

#### 3.3.1. Primary Outcome

At baseline, the mean score on the impact of bladder-related symptoms (UIQ-7) was 33.6 points (95%CI 30.9; 36.2), which improved significantly at the intervention end (mean change: 8.8, 95%CI 4.7; 12.9, *p* < 0.001; Table 3). The response rate to treatment was 57.3% (95%CI 54.8; 59.7) (*p* < 0.001), according to the MCIC reported by Barber et al. [34].

Older participants reported worse UIQ-7 scores at baseline, with an increase of 0.5 points (95%CI 0.2; 0.9) per extra year above the mean cohort age (*p* = 0.001). BMI and parity did not significantly influence baseline scores, and no covariate impacted the recovery trajectory (Supplementary Table S4).

#### 3.3.2. Secondary Outcomes

The impact of overall pelvic floor symptoms (including bladder, bowel, or vaginal symptoms) on daily activities was reduced by treatment end (PFIQ-7: 17.9, 95%CI 5.9; 29.9; Table 3). Older participants reported a higher impact at baseline but recovered similarly to their younger counterparts (Supplementary Table S4). Neither BMI nor parity significantly influenced baseline or recovery trajectories (Supplementary Table S4).

Regarding mental health, 42% of participants (137/326) reported relevant anxiety symptoms [30] (GAD-7 ≥ 5: 9.0, 95%CI 8.3; 9.7), while 26.4% of participants (86/326) reported depression scores above the threshold [31] (PHQ-9 ≥ 5: 10.6, 95%CI 9.6; 11.6) at baseline. A significant correlation was observed between higher baseline anxiety or depression and higher UIQ-7 (correlation coefficient r(326) = 0.241 and 0.233, respectively; both *p* < 0.001). Significant reduction in mental symptoms was reported by those participants at program-end, with 59.7% (95%CI 55.6; 63.7, *p* < 0.001) reaching the MCIC for anxiety (GAD-7) [36] and 57.1% (95%CI 52.2; 61.9, *p* = 0.01) reaching the MCIC for depression (PHQ-9) [37]. None of the covariates influenced recovery trajectories (Supplementary Table S4).

Among all employed participants, 41.7% (113/271) reported impairment in overall productivity, of whom only 24 experienced absenteeism (8.9% of the total employed population). Overall, productivity losses were significantly reduced at the program end, with 64.2% (95%CI 60.0; 68.3; *p* = 0.01) of participants reaching the MCIC for overall productivity impairment (WPAI overall) [38]. Productivity recovery was not influenced by age, BMI, or parity (Supplementary Table S4).

A total of 84% of participants (*n* = 274/326) reported intention to seek additional healthcare to address their condition at baseline, which was reduced by 55.9% from 4.9/10 (95%CI 4.5; 5.2) to 2.1 (95%CI 1.5; 2.8) at study end (*p* < 0.001). Older participants reported greater intent to seek additional healthcare at baseline, at a mean of 0.04 extra points (95%CI 0.0; 0.1) per extra year above the cohort mean age (*p* = 0.024), but reported a reduction in their intent similar to younger counterparts (Supplementary Table S4). No other covariate influenced the recovery trajectory (Supplementary Table S4).

### 3.4. Adverse Events

Across the intervention, no serious adverse events were reported. Adverse events potentially related to the intervention (e.g., pelvic pain and pressure, urinary infection, ache, and cramping, etc.) were reported by 2.1% (7/326) of the cohort, while adverse events non-related to the intervention (e.g., unrelated sickness) were reported by 2.8% (9/326) (Supplementary Table S5).

### 3.5. Engagement Outcomes

Treatment ran over an average of 10.3 (SD 5.0) weeks, with participants doing an average of 18.0 (SD 11.4) pod-associated sessions. The majority of participants engaged with the educational content (95.7%, 312/326) by reading, on average, 21.0 (SD 12.9) educational articles. Bi-directional communication was high across the program, with an average of 38.8 (SD 36.1) mean total number of touchpoints. Participants reported high satisfaction with the program (8.9/10, SD 1.8).

## 4. Discussion

### 4.1. Main Findings

This study provides real-world evidence to support the feasibility and safety of this pelvic DCP to provide care to participants with UI. Significant improvement was observed in the impact of stress, urgency, and mixed UI on the participants’ daily living (UIQ-7), with an overall response rate of 57.3% (95%CI 54.8–59.7). This improvement was accompanied by a reduction in the impact of other pelvic conditions (PFIQ-7 reduction: 17.9, 95%CI 5.9; 29.9). Alongside the pelvic health improvement, mental health distress was significantly reduced, with 59.7% and 57.1% of responders (i.e., reaching MCID) for anxiety and depression, respectively. Additional benefits were observed in the productivity domain, where 64.2% (95%CI 60.0; 68.3) attained significant recovery after the intervention. The success of this pelvic DCP was further reinforced by the 55.9% reduction in the intention to seek additional healthcare services as well as high satisfaction levels (8.9/10, SD 1.8).

### 4.2. Comparison with the Literature

The intervention attained a very high completion rate (81.0%), higher than those reported in previous in-person [39] and digital studies [19,22] and within the range of another digital study [20].

This study cohort had a mean age of 44.8, similar to the reported by previous digital studies [18], and younger than the reported for other UI studies [19,23,24]. The distribution of the UI types followed the description for the US population [1,40], although with a larger concentration of stress UI (73.3%, 239/326), possibly associated with the younger portion of the study cohort (37.1%) [41]. Significant risk factors associated with UI development were reported by this study cohort at baseline: older age (62.9% of participants above 40 years), higher BMI (39.3% obese or morbidly obese), being parous (92%), and reporting the condition for more than 6 months (85.6%) [9,41]. Non-completers were younger and had higher BMI levels than those completing the program, which is consistent with previous observations [19,23]. The assessment of adherence to PFMT interventions is not standardized across the literature, nor are the frequency and length of such interventions for UI, which results in high heterogeneity between reports, hampering direct comparisons [21,22,23]. Herein, we report an average of 18.0 (SD 11.4) pod-associated sessions during 10.3 (SD 5.0) weeks, which, although slightly below the default recommendation, is on par with that reported by other digital interventions [23]. Nevertheless, this observation seemed to not compromise clinical improvements, in line with previously reported nonlinearity between outcome improvement and adherence [25]. In fact, Keyser et al. [23] have suggested that fewer exercise sessions engagement might provide adequate neuromuscular re-education to positively affect functional PFM performance. Moreover, very high adherence to the educational component (articles read: 21.0, SD 12.9) was observed herein. High engagement with education is critical, considering the empowering effect on patient self-management and the lack of pelvic health literacy within the general population [10].

At baseline, the impact of the condition on activities of daily living (UIQ-7) was 33.6/100 points (95%CI 30.9; 36.2). By the end of the intervention, the mean improvement was 8.8 points (95%CI 4.7; 12.9) (*p* < 0.001), with a response rate of 57.3% (95%CI 54.8; 59.7) (*p* < 0.001). These improvements are within the range of those observed in previous studies [20,23,42], with a similar response rate to the reported in-person [21] and digital intervention studies [23]. Older participants reported worse baseline scores, which is consistent with previous reports [43]. Interestingly, despite being frequently associated with a poorer prognosis, neither age, BMI, nor parity significantly influenced UIQ-7 reduction. A similar trend was observed with the concomitant recovery on the impact of overall pelvic floor symptoms (including bladder, bowel, or vaginal symptoms) on daily activities (mean change: 17.9, 95%CI 5.9; 29.9), where older participants reported a worse baseline, but recovery trends were not influenced by any covariates. These findings are in line with a previous trial that compared telerehabilitation with usual care [19].

Importantly, the adverse event rate was very low across the intervention, and all the reported events were minor. The nature of the reported adverse events and the incidence rates were similar to the previously reported in-person pelvic rehabilitation [7,21] and lower compared to other digital interventions [23]. This result suggests the safety of our intervention, a crucial aspect to ensure, particularly in remote rehabilitation settings as described herein.

Improvements in pelvic health might have supported the recovery reported within the mental health domain. It is known that mental distress can impact the odds of UI recovery [44], despite most studies focusing solely on urinary-related outcomes [7]. Herein, 42% and 26% of participants reported at baseline relevant anxiety and depression symptoms [31], respectively. These followed the trend reported by Felde et al., who reported a two-fold increased risk of depression or anxiety among patients with mixed urinary incontinence [45]. Importantly, despite the correlation between mental distress and worse UIQ-7 scores at baseline (*p* < 0.001), 59.7% of patients (95%CI 55.6; 63.7, *p* < 0.001) reached the MCIC for anxiety (GAD-7) [36] and 57.1% of patients (95%CI 52.2; 61.9, *p* = 0.01) reached the MCIC for depression (PHQ-9) [37]. These reductions were observed regardless of age, BMI, or parity.

Work productivity impairment is frequently associated with UI conditions, translated more frequently into presenteeism than absenteeism [46], in some cases demanding a complete revamp of patients’ professional lives to best cope with the limitations imposed by the condition [2]. Herein, this same trend was observed at baseline, with 42% of the cohort reporting presenteeism. Significant improvements were observed in overall productivity impairment with 64.2% (95%CI 60.0; 68.3; *p* = 0.01) of patients responding to the intervention considering an MCIC of 7 [38,46], suggesting a positive impact on productivity and associated financial burden.

All the aforementioned outcomes explain the significant reduction in the intent to seek additional healthcare (55.9%), which, alongside the high satisfaction levels (8.9/10, SD 1.8), reinforces the potential of this DCP as a viable care delivery option to provide timely access to care, mitigating the shortages of healthcare providers, while empowering patients with urinary conditions to improve their pelvic health and overall quality of life.

### 4.3. Strengths and Limitations

The major strength of this study is the novelty of the approach—a completely remote, multimodal DCP combining PFMT with real-time biofeedback and condition-specific education based on a biopsychosocial framework. Biofeedback data and PT management (including on-demand communication) fostered individualized care and safety. While it is conceivable for individuals to perform a PFMT program independently, the involvement of a qualified health professional is a major strength for continuous correct training, providing clinical assessment, ongoing monitoring, and the delivery of personalized, evidence-based programs. The expertise of PTs is crucial to navigating the intricacies of the diverse clinical presentations and individual needs, addressing not only the symptoms but also the underlying factors contributing to the condition through effective interventions, as previously reported [7,47]. The inclusion of diverse UI types enhances generalizability since most studies focus solely on stress UI [18,20,22]. Other strengths encompass the large sample size, multiple domain outcomes, and objective measures to assess engagement (instead of self-registration [20]). Limitations include (a) study design, which is observational and lacks a control group; (b) lack of physical examination, which, although recommended by guidelines, might not be attained in an in-person context but can be facultative as per COVID pandemic lessons [48]; and (c) lack of objective measures of PFM function at baseline and follow-up. Considering the real-world context of the study, (d) we cannot rule out the possibility that medications (e.g., anticholinergics, hormonal therapies) or pessaries might have been used by some patients and influenced outcomes. Future studies should assess the long-term maintenance of the observed improvements. Nevertheless, we believe this study adds to the body of evidence with insights into the feasibility, safety, and outcomes of digital intervention in UI management. This knowledge advocates for the further planning and conduct of future randomized controlled trials.

## 5. Conclusions

This study shows the feasibility and safety of a completely remote DCP with biofeedback managed asynchronously by a physical therapist to reduce UI-related symptoms in a real-world setting. Significant improvements were observed in the UI burden across all outcome domains (daily activities, mental health, productivity impairment, and intention to seek additional care). Together, these findings advocate for the exploration of this care delivery option to escalate access to proper and timely UI care, empowering patients with urinary conditions to improve their pelvic health and overall quality of life.

## Figures and Tables

**Table 1:** Study secondary outcomes.

Outcome	Measure
**Engagement metrics**	
Program completion rate	% of individuals completing the program
Exercises engagement	Total number of exercise sessions performed with the sensor. Note: Sessions performed without the intravaginal sensor were not accounted for.
Education engagement	Number of educational pieces read
Touchpoints between the patient and the PT	Number of touchpoints, either by video, phone calls, or text messages, initiated by either the patient or PT
Satisfaction	“On a scale from 0 to 10, how likely are you to recommend Bloom’s programs to a friend or family member?”
**Clinical outcomes**	
Pelvic floor symptoms impact on daily living	Pelvic Floor Impact Questionnaire—short form 7 (PFIQ-7); (scores 0–300). Higher scores denote worse outcomes.
Mental health	Anxiety: Generalized Anxiety Disorder 7-item scale (GAD-7) (scores 0–21) [30], in the past 2 weeks. Depression: Patient Health 9-item Questionnaire (PHQ-9) (scores 0–27) [31], in the past 2 weeks. A cut-off threshold ≥ 5 indicates at least mild anxiety and depression, respectively.
Work productivity and activity impairment	Collected within the employed population: Work Productivity and Activity Impairment (WPAI) for general health to assess overall work impairment (WPAI overall), presenteeism (WPAI work), and absenteeism (WPAI time) [29]. Scores range from 0 to 100%, with higher scores indicating higher impairment.
Intent to seek additional healthcare	“How likely are you to seek additional healthcare interventions for this condition?” (0—not at all likely to 10—extremely likely).

**Table 2:** Baseline characteristics of study participants: entire cohort (*n* = 326).

Characteristic	Entire Cohort (*n* = 326)
**Age (years), mean (SD)**	44.8 (9.1)
**Age categories (years), *n* (%):**	
<25	1 (0.3)
25–40	120 (36.8)
41–54	149 (45.7)
≥55	56 (17.2)
**Gender, *n* (%)**	
Female	325 (99.7)
Prefer not to specify	1 (0.3)
**BMI (kg/m^2^), mean (SD)**	29.7 (7.0)
**BMI categories (kg/m^2^), *n* (%):**	
Underweight (<18.5)	3 (0.9)
Normal (18.5–25)	90 (27.6)
Overweight (≥25–30)	105 (32.2)
Obese (≥30–40)	100 (30.7)
Morbidly obese (>40)	28 (8.6)
**Employment status, *n* (%):**	
Employed full-time	275 (84.4)
Employed part-time	27 (8.3)
Unemployed (not working or retired)	21 (6.4)
Prefer not to specify	3 (0.9)
**Education level, *n* (%):**	
High school diploma or less than high school	22 (6.7)
Some college	85 (25.1)
Bachelor’s degree	149 (45.7)
Graduate degree	66 (20.2)
Prefer not to specify	4 (1.2)
**Race/ethnicity, *n* (%):**	
Asian or Pacific Islander	15 (4.6)
Black or African American	13 (4.0)
Hispanic or Latino	31 (9.5)
White or Caucasian	249 (76.4)
American Indian or Alaska Native	5 (1.5)
Multi-racial or bi-racial	7 (2.1)
Prefer not to specify	5 (1.5)
Not listed	1 (0.3)
**Urinary Incontinence Type**	
Mixed urinary incontinence	64 (19.6)
Stress urinary incontinence	239 (73.3)
Urgency urinary incontinence	23 (7.1)
**Acuity, *n* (%):**	
Less than 6 months	29 (8.9)
More than 6 months	280 (85.6)
Not available	17 (5.2)
**Parity ^a^, mean (SD)**	2.0 (1.2)
**Parity ^a^, *n* (%):**	
Nulliparous	22 (8.0)
1–2	178 (64.5)
3–5	75 (27.2)
>5	1 (0.4)

Abbreviations: BMI, body mass index. Notes: ^a^: *n* = 276.

**Table 3:** Model estimates of clinical outcome measures following an intent-to-treat approach.

Outcome, Mean (95%CI)	*n*	Baseline	End-of-Program	Mean Change
UIQ-7	311	33.6 (30.9;36.2)	24.8 (21.0;28.5)	8.8 (4.7;12.9)
PFIQ-7	313	60.1 (52.8;67.5)	42.3 (32.0;52.6)	17.9 (5.9; 29.9)

Abbreviations: PFIQ-7, Pelvic Floor Impact Questionnaire—short form 7; UIQ-7, Urinary Impact Questionnaire—short form 7.

## Data Availability

The data presented in this study are available on request from the corresponding author. The data are not publicly available due to privacy restrictions.

## Supplementary Materials

The following supporting information can be downloaded at: https://www.mdpi.com/article/10.3390/healthcare12020141/s1, Table S1: Details of the intervention protocol; Table S2: Baseline characteristics between study completers (*n* = 264) and non-completers (*n* = 62); Table S3: Unconditional model estimations and model fit: intention-to-treat approach; Table S4: Conditional model with age, body mass index, and parity as covariates; Table S5: Adverse events reported across the intervention, classified by condition and non-condition-related.
