# Supplementary material for: Digital Care Program for Urinary Incontinence in Females: A Large-Scale, Prospective, Cohort Study
Source: Healthcare (Basel). 2024 Jan 8;12(2):141. doi: 10.3390/healthcare12020141 (PMC10815799; doi:10.3390/healthcare12020141)
Supplement: Supplementary file 1 [file healthcare-12-00141-s001.zip › healthcare-2777519-supplementary.pdf]

Supplementary Materials

Digital Care Program for Urinary Incontinence in Females: a large-scale, prospective, cohort study

Dora Janela, Anabela C Areias, Maria Molinos, Robert G Moulder, Ivo Magalhães, Virgílio Bento, Marta Cardeano, Vijay Yanamadala, Fernando Dias Correia, Jennesa Atherton, Fabíola Costa

Supplementary Table S1. Details of the intervention protocol.

Intervention Component	Main goals	Precautions	Description
Education:<br>Initial preparation before PFMT<br>Ongoing education	Improve patient's literacy on the condition<br><br>Introduction to the basic technical aspects needed before initiating PFMT (contraction and relaxation techniques and breathing)<br><br>Facilitate the adoption of healthy lifestyle habits<br><br>Empowering patient's self-management of the condition		Main topics included on the initial preparation before PFMT:<br>Basic anatomy and physiology of the pelvic floor<br>The role and importance of exercise<br>How to contract and relax the PFM<br>Diaphragmatic breathing (which was adopted during PFMT)<br>How to insert, use, and sanitize the intravaginal sensor<br><br>Ongoing education comprise the following topics:<br>Pathophysiology of the urinary condition<br>Contributing factors for the urinary condition<br>Relaxation techniques<br>Lifestyle modification advice and strategies<br>Bladder retraining and toileting techniques<br><br>These aspects were introduced after the completion of the initial preparation until the program-end.
Pelvic floor muscle Training (PFMT):<br>In isolation<br>In combination with functional exercises	Improve pelvic floor power, endurance, speed, and adaptability<br><br>Improve coordination between the PFM and surrounding muscle groups (e.g.,	Avoid Valsava maneuver	Four categories of exercises were included, (all performed in a hooklying position):<br>1) Strength, requiring a maximum contraction of PFM;<br>2) Endurance, requiring a sustained contraction of the PFM at a target level;<br>3) Control, requiring contractions and relaxations at a target level ;<br>4) Agility, requiring rapid contractions followed by a relaxation at a target level to

abdominal muscles, hip muscles)	simulate the activity of the PFM in reflex contractions, such as during cough.
Improve pelvic girdle and pelvic floor mobility	PFMT was combined with the following functional exercises (performed in different positions, i.e., hooklying and quadruped):<br>- abdominal strengthening exercises<br>- lower limb strengthening exercises<br><br>Pelvic floor muscle exercises were initiated after the initial education. Afterwards, exercises were gradually progressed through the combination of PFMT with functional exercises (after at least 3 sessions until the program-end).<br><br>Example of a session:<br>1. Strength for 3 reps<br>2. Control - Contraction 6:4 seconds on/off for 59 seconds (116 targets*)<br>3. Hold - Contraction 2:2 seconds on/off for 3 reps<br>4. Agility - Contraction for 10 reps every 1 second<br>5. Bridge with Pelvic Floor Contractions for 5 reps<br>6. Hold - Contraction 3:3 seconds on/off for 3 reps<br>7. Diaphragmatic Breathing for 10 reps

Note: Parameters of each exercise were adjusted by the physical therapist, such as the number of repetitions, sets, and duration of contractions and relaxations, according to the patient's condition. Participants were recommended to complete at least 3 sessions per week.
*The target level refers to the degree of contraction or relaxation prescribed by the physical therapist for a given exercise.

Supplementary Table S2. Baseline Characteristics between Study Completers (N=264) and Non-Completers (N=62).

Characteristic	Completers (N=264)	Non-completers (N=62)	P
**Age (years), mean (SD)**	45.3 (9.1)	42.7 (9.1)	*.041*
**Age categories (years), N (%):**			*.030*
<25	0 (0)	1 (1.6)	
25-40	93 (35.2)	27 (43.5)	
41-54	120 (45.5)	29 (46.8)	
≥55	51 (19.3)	5 (8.1)	
**Gender, N (%)**			.627
Female	263 (99.6)	62 (100)	
Prefer not so to specify	1 (0.4)	0 (0)	
**BMI (kg/m²), mean (SD)**	29.2 (6.7)	31.7 (7.9)	*.009*
**BMI categories (kg/m²), N (%):**			.072
Underweight (<18.5)	3 (1.1)	0 (0)	
Normal (18.5-25)	79 (29.9)	11 (17.7)	
Overweight (≥25-30)	84 (31.8)	21 (33.9)	
Obese (≥30-40)	80 (30.3)	20 (32.3)	
Morbidly obese (>40)	18 (6.8)	10 (16.1)	
**Employment status, N (%):**			.488
Employed full-time	219 (83.0)	56 (90.3)	
Employed part-time	24 (9.1)	3 (4.8)	
Unemployed (not working or retired)	18 (6.8)	3 (4.8)	
Prefer not to specify	3 (1.1)	0 (0)	
**Education level, N (%):**			.846
High school diploma or less than high school	19 (7.2)	3 (4.8)	
Some college	68 (25.8)	17 (27.4)	
Bachelor's degree	123 (46.6)	26 (41.9)	
Graduate degree	51 (19.3)	15 (24.2)	
Prefer not to specify	3 (1.1)	1 (1.6)	
**Race/ethnicity, N (%):**			.218
Asian or Pacific Islander	12 (4.5)	3 (4.8)	
Black or African American	7 (2.7)	6 (9.7)	
Hispanic or Latino	28 (10.6)	3 (4.8)	
White or Caucasian	201 (76.1)	48 (77.4)	
American Indian or Alaska Native	5 (1.9)	0 (0)	

Multi-racial or bi-racial	6 (2.3)	1 (1.6)	
Prefer not to specify	4 (1.5)	1 (1.6)	
Not listed	1 (0.4)	0 (0)	
**Urinary Incontinence Type, N (%):**			.721
Mixed urinary incontinence	54 (20.5)	10 (16.1)	
Stress urinary incontinence	192 (72.7)	47 (75.8)	
Urgency urinary incontinence	18 (6.8)	5 (8.1)	
**Acuity, N (%):**			.368
Less than 6 months	21 (8.0)	8 (12.9)	
More than 6 months	228 (86.4)	52 (83.9)	
Not available	15 (5.7)	2 (3.2)	
**Parity[a], mean (SD)**	2.0 (1.2)	1.8 (1.0)	.271
**Parity[a], N (%):**			.645
Nulliparous	16 (7.1)	6 (11.8)	
1-2	145 (64.4)	33 (64.7)	
3-5	63 (28.0)	12 (23.5)	
>5	1 (0.4)	0 (0)	
**Clinical scores, mean (SD)**			
UIQ-7	33.2 (25.1)	26.7 (25.8)	.066
PFIQ-7	60.9 (70.8)	44.6 (62.1)	.098
Medications consumption (yes), N (%)	5 (1.9)	2 (3.2)	.515
Intent to seek additional healthcare	4.2 (3.2)	4.7 (2.9)	.269
GAD-7 ≥ 5[b]	9.0 (4.2)	9.1 (4.0)	.876
GAD-7	4.2 (4.9)	4.9 (5.1)	.309
PHQ-9 ≥ 5[c]	10.0 (4.4)	12.8 (5.7)	.027
PHQ-9	2.9 (4.9)	3.9 (6.5)	.145
WPAI overall >0[d]	25.2 (15.7)	32.8 (22.5)	.124
WPAI overall[e]	10.2 (15.9)	15.5 (22.5)	.111
WPAI work >0[f]	24.0 (14.3)	31.2 (22.8)	.145
WPAI work[e]	9.4 (14.7)	14.7 (22.1)	.098
WPAI time >0[g]	14.1 (12.2)	8.8 (7.6)	.328
WPAI time[e]	1.2 (5.2)	1.0 (3.7)	.821

Abbreviations: BMI, body mass index; GAD-7, Generalized Anxiety Disorder 7-item scale; PFIQ-7, Pelvic Floor Impact Questionnaire - short form 7; PHQ-9, Patient Health 9-item questionnaire; UIQ-7, Urinary Impact Questionnaire - short form 7; WPAI, Work Productivity and Activity Impairment Questionnaire.

Notes: a: Completers: N= 225 and Non-completers N=51; b: Completers: N= 107 and Non-completers N=30; c: Completers: N= 68 and Non-completers N=18; d: Completers: N= 88 and Non-completers N=25; e: Completers: N= 218 and Non-completers

N=53; f: Completers: N= 85 and Non-completers N=25; g: Completers: N= 18 and Non-completers N=6.

Supplementary Table S3. Unconditional model estimations and model fit: intention-to-treat approach.

		Intercept		Slope		Fit				
Outcome	N	Mean (SD)	*p*	Mean (SD)	*p*	Chi-sq (df)	*p*	CFI	RMSEA	SRMR
UIQ-7 >0	311	33.6 (10.5)	<.001	-0.9 (0)	<.001	0.012 (1)	0.914	1.000	0.000	0.008
PFIQ-7 >0	313	60.1 (32.6)	<.001	-1.8 (4.9)	.004	0.001 (1)	0.977	1.000	0.000	0.001
Intent to seek additional healthcare >0	274	4.9 (1.5)	<.001	-0.3 (0.2)	<.001	22.807 (1)	0.000	0.702	0.282	0.119
Intent to seek additional healthcare	326	4.1 (1.4)	<.001	-0.2 (0.1)	<.001	10.359 (1)	0.001	0.888	0.169	0.069
GAD-7 ≥5	137	9.0 (3.8)	<.001	-0.3 (0.4)	<.001	4.752 (1)	0.029	0.939	0.165	0.073
GAD-7	326	4.3 (4.1)	<.001	-0.1 (0.3)	.003	0.016 (1)	0.899	1.000	0.000	0.002
PHQ-9 ≥5	86	10.6 (5.0)	<.001	-0.4 (0.5)	<.001	0.483 (1)	0.487	1.000	0.000	0.058
PHQ-9	326	3.0 (4.8)	<.001	-0.1 (0.3)	.162	0.215 (1)	0.643	1.000	0.000	0.010
WPAI Overall >0	113	24.7 (7.4)	<.001	-0.8 (1.6)	.004	9.230 (1)	0.002	0.600	0.254	0.133
WPAI Overall	271	11.1 (9.1)	<.001	-0.2 (0.4)	.108	0.001 (1)	0.972	1.000	0.000	0.001
WPAI Work >0	110	23.8 (9.2)	<.001	-0.8 (1.6)	<.001	8.213 (1)	0.004	0.761	0.240	0.126
WPAI Work	271	10.3 (10.3)	<.001	-0.2 (1.0)	.169	0.033 (1)	0.855	1.000	0.000	0.004
WPAI Time >0	24	7.2 (0)	.079	-0.7 (0.1)	.097	9.680 (1)	0.002	0.000	0.472	0.315
WPAI Time	271	1.1 (0.1)	<.001	0 (0)	.875	0.056 (1)	0.813	1.000	0.000	0.008

Abbreviations: GAD-7, Generalized Anxiety Disorder 7-item scale; PFIQ-7, Pelvic Floor Impact Questionnaire - short form 7; PHQ-9, Patient Health 9-item questionnaire; UIQ-7, Urinary Impact Questionnaire - short form 7; WPAI, Work Productivity and Activity Impairment Questionnaire.

Note: Model fitness was assessed through chi-squared test, root mean square error of approximation (RMSEA), confirmatory fit index (CFI), and standardized root mean square residual (SRMR), according to the criteria: CFI = close to 0.95; RMSEA = close to 0.06 and SRMR = close to 0.08. Significant p-values and model fit values indicating good model fit are presented in italic.

Supplementary Table S4. Conditional model with age, body mass index and parity as covariates.

**Outcome**	**Age**				**BMI**				**Parity**				**FIT**				
Mean (95% CI)	Intercept	P	Slope	P	Intercept	P	Slope	P	Intercept	P	Slope	P	Chi-sq (df)	*P*	CFI	RMSEA	SRMR
UIQ-7 >0	0.5 (0.2;0.9)	0.001	0 (-0.1;0)	0.183	0.3 (-0.1;0.7)	0.187	0 (-0.1;0)	0.644	0.5 (-1.8;2.7)	0.687	0.2 (-0.3;0.6)	0.536	0.554 (4)	0.968	1.000	0.000	0.011
PFIQ-7 >0	1.2 (0.3;2.1)	0.007	0 (-0.2;0.1)	0.537	0.2 (-0.9;1.3)	0.668	0 (-0.2;0.2)	0.925	2.2 (-4.3;8.7)	0.509	0.3 (-1.1;1.7)	0.694	1.434 (4)	0.838	1.000	0.000	0.016
Intent to seek additional healthcare >0	0.04 (0;0.1)	0.024	0 (0;0)	0.707	0 (-0.1;0)	0.352	0 (0;0)	0.757	0 (-0.3;0.3)	0.777	0 (-0.1;0.1)	0.864	19.733 (4)	0.001	0.732	0.129	0.079
Intent to seek additional healthcare	0.04 (0;0.1)	0.029	0 (0;0)	0.641	0 (-0.1;0)	0.567	0 (0;0)	0.791	-0.2 (-0.5;0.1)	0.225	0 (0;0.1)	0.876	10.710 (4)	0.030	0.899	0.078	0.047
GAD-7 ≥5	0 (-0.1;0.1)	0.899	0 (0;0)	0.908	0 (-0.1;0.1)	0.466	0 (0;0)	0.569	-0.3 (-0.9;0.3)	0.273	0.1 (0;0.2)	0.169	6.415 (4)	0.170	0.953	0.072	0.058
GAD-7	0 (-0.1;0.1)	0.910	0 (0;0)	0.363	0.1 (0;0.2)	0.040	0 (0;0)	0.585	-0.4 (-0.8;0)	0.075	0 (-0.1;0.1)	0.503	3.020 (4)	0.555	1.000	0.000	0.016
PHQ-9 ≥5	0 (-0.1;0.1)	0.726	0 (0;0)	0.479	0.1 (-0.1;0.2)	0.207	0 (0;0)	0.470	0.6 (-0.4;1.6)	0.230	0 (-0.1;0.2)	0.652	6.446 (4)	0.168	0.878	0.094	0.083
PHQ-9	0 (0;0.1)	0.280	0 (0;0)	0.796	0.1 (0.1;0.2)	0.001	0 (0;0)	0.081	-0.2 (-0.6;0.3)	0.503	0 (-0.1;0.1)	0.744	6.746 (4)	0.150	0.982	0.050	0.029
WPAI Overall >0	-0.4 (-0.9;0)	0.039	0 (-0.1;0.1)	0.817	-0.2 (-0.5;0.2)	0.350	0 (-0.1;0.1)	0.912	-3.5 (-6.3;-0.8)	0.012	-0.2 (-0.6;0.3)	0.438	13.578 (4)	0.009	0.705	0.130	0.072
WPAI Overall	-0.1 (-0.3;0.1)	0.325	0 (-0.1;0)	0.336	0.2 (-0.1;0.4)	0.197	0 (-0.1;0)	0.260	-2.6 (-4.7;-0.6)	0.012	0 (-0.2;0.2)	0.859	3.928 (4)	0.416	1.000	0.000	0.035
WPAI Work >0	-0.2 (-0.6;0.1)	0.187	0 (-0.1;0.1)	0.994	-0.1 (-0.4;0.2)	0.563	0 (0;0.1)	0.677	-3.4 (-5.9;-0.9)	0.008	-0.1 (-0.5;0.3)	0.562	10.071 (4)	0.039	0.829	0.104	0.072
WPAI Work	0 (-0.2;0.2)	0.737	0 (-0.1;0)	0.070	0.2 (0;0.5)	0.077	0 (-0.1;0)	0.060	-2.7 (-4.5;-0.9)	0.003	0 (-0.2;0.2)	0.847	1.552 (4)	0.817	1.000	0.000	0.015
WPAI Time*	-0.1 (-0.2;0)	0.015	0 (0;0)	0.560	0 (-0.1;0)	0.230	0 (0;0)	0.210	0 (-0.7;0.6)	0.977	0 (-0.1;0.1)	0.525	4.111 (4)	0.391	0.977	0.010	0.043

Abbreviations: BMI, body mass index; GAD-7, Generalized Anxiety Disorder 7-item scale; PFIQ-7, Pelvic Floor Impact Questionnaire - short form 7; PHQ-9, Patient Health 9-item questionnaire; UIQ-7, Urinary Impact Questionnaire - short form 7; WPAI, Work Productivity and Activity Impairment Questionnaire.

Note: Model fitness was assessed through chi-squared test, root mean square error of approximation (RMSEA), confirmatory fit index (CFI), and standardized root mean square residual (SRMR), according to the criteria: CFI = close to 0.95; RMSEA = close to 0.06 and SRMR = close to 0.08. Significant p-values and model fit values indicating good model fit are presented in bold.
*The conditional model for WPAI Time in filtered cases did not converge.

Supplementary Table S5. Adverse events reported across the intervention classified by condition and non-condition related.

Type	Occurrence (#N)	Description
Intervention-related	1	Right sided pelvic pain and pressure
	1	Urinary Tract Infection
	2	Muscle soreness/cramping
	1	Worsening leak episodes
	1	Pain inserting the pod
	1	Yeast infection
Non-intervention related	6	Unrelated medical illness (including COVID-19 infection
	1	Surgical procedure for lysis of bladder scar
	1	Unrelated surgery to another body area
	1	Unrelated injury to another body area
